# Qualitative content analysis of reactivity effects and feasibility of ecological momentary assessments of suicide-related thoughts and behaviors in the long-term and in suicidal crises

**DOI:** 10.3389/fpsyt.2026.1744947

**Published:** 2026-03-06

**Authors:** Lena Spangenberg, Cora Spahn, Jana Serebriakova, Thomas Forkmann, Heide Glaesmer

**Affiliations:** 1 Department of Medical Psychology and Medical Sociology, University of Leipzig, Leipzig, Germany; 2 Department of Clinical Psychology and Psychotherapy, Institute of Psychology, University of Duisburg-Essen, Essen, Germany

**Keywords:** ecological momentary assessment, feasibility, qualitative content analysis, reactivity, suicide-related thoughts and behaviors

## Abstract

**Background:**

This study explored participant experiences with ecological momentary assessment (EMA) in the context of suicidal thoughts and behaviors (STBs).

**Methods:**

16 participants of a long-term EMA study (with varying STB occurrence during the study and low vs. high compliance) were interviewed on reactivity effects and feasibility of EMA. Qualitative content analysis was performed using an inductive-deductive approach and consensual coding.

**Results:**

Reactivity to EMA was reported by some participants, with suicidal thoughts occasionally intensifying/being triggered by survey prompts. Importantly, no evidence indicated that EMA triggered suicidal actions. However, the burden increased over time for some, calling for more personalized monitoring durations. EMA’s feasibility during acute suicidal crises was questioned due to reduced ability and willingness to respond.

**Conclusions:**

Long-term EMA monitoring after psychiatric discharge was perceived as feasible and beneficial. Selection bias and the lack of quantitative validation limit generalizability. Findings underscore the value of mixed-methods approaches and participatory protocol design.

## 1 Introduction

In suicide research, real-time data collection methods such as ecological momentary assessments (EMA) have been increasingly used in the past decade (1, 2). Besides general benefits of EMAs (i.e., high ecological validity, reduction of recall bias) (3), their particular importance in research on suicide-related thoughts and behaviors (STBs) lies in their ability to capture short-term fluctuations in STBs as well as in related proximal risk-factors. EMAs can therefore improve our understanding of the temporal dynamics, as well as the associated or preceding states, thoughts, and contextual factors related to STBs (4).

EMA studies have indeed proven to provide insights in the temporal dynamics of suicidal thoughts (5, 6) and its short-term predictors such as interpersonal variables (7), sleep (8, 9) or affective states (10). When discussing findings, researchers often claim that real-time data collection methods will pave the way for intervening in real-time (ecological momentary interventions [EMI], for using just-in-time adaptive interventions [JITAIs]) (11, 12) and might aid in monitoring STB risk and consequently prevent suicide attempts and suicides (2).

The integration of EMA/EMI into clinical practice remains, however, generally challenging (13). There is great enthusiasm for the possibility of intervening in times of acute risk, but the scientific evidence lags behind (14). Several methodological, practical and ethical aspects contribute to this lack of evidence:

It is particularly difficult for studies to identify individual moments at risk due to the dynamic nature of STBs (15, 16) and their low base rate (17, 18). While the high temporal resolution of EMA studies matches the dynamic nature of STBs and captures clinically relevant periods of time (18), the low base rate of STBs and specifically of suicidal behavior poses a major problem for EMA studies (19). Indeed, available EMA research largely focuses on suicidal ideation which is more prevalent in clinical and non-clinical populations and easier to study than suicidal behavior (2, 20). Studies aiming to gain insights in the unfolding of a suicidal crisis and suicidal behavior need to focus on high-risk populations and on high-risk periods (i.e., persons after a suicide attempt, the post-discharge period) (21–23) to ensure a sufficient base rate for sound statistical modelling and to zoom into a suicidal crisis. In addition, studies need to either examine a considerably large sample or require a longer study duration to ensure adequate power (i.e., a certain number of events) (18, 19).

Yet, the feasibility of such studies targeting high-risk populations and times of particular high STB risk is not well investigated and such studies are ethically challenging (24). While the feasibility of EMA study designs has been mainly positively evaluated so far (e.g, (25, 26)), the majority of studies has been limited to the study of suicide ideation and the follow-up periods have been of limited duration (median length 14 days, range 4 to 60 days; (2)). Findings from the few available studies conducted in populations at high risk of STBs such as patients after discharge from a psychiatric ward often suffer from low response (i.e., eligible persons willing to participate in the study), attrition (i.e., participants completing the whole EMA period), and compliance rates (i.e., the ratio of completed assessments over the maximum assessments per protocol) (23). For instance, two studies reported compliance rates of 21% (27) and 14 to 16% only (23) within a 3-week or 8-week timeframe of EMA following discharge. Studies with longer observation intervals have also shown low compliance rates (e.g. 17.6% in 3–6-month EMA study in outpatients (28)). These findings are in line with meta-analytic evidence that lower EMA compliance tends to be associated with higher symptom burden and increasing study duration (29, 30). Most importantly, the low compliance might be indicative of a questionable feasibility of study protocols longer than two or three weeks, that are required to capture the unfolding of a suicidal crisis with a certain likelihood. Beyond this, it remains unclear how the specific needs of the target populations can be addressed in study protocols and whether EMAs are a useful and feasible approach for investigating suicidal crises in real time from the perspective of the persons experiencing it.

Besides indicating low feasibility and high burden of long study protocols, low compliance raises concerns related to reactivity effects as well. *Measurement reactivity* is defined as changes in the underlying construct (e.g., iatrogenic effects such as exacerbation of symptoms), in behavior (e.g., avoidance of activities or adaption of daily routines), and in response behavior (e.g., change in use of response scales during the participation period) due to intensively repeated assessments (31). While the number of missing data is usually not problematic for statistical modelling or power (specifically if the study protocol has assumed a certain amount of missing data *a priori* (29)), it is unclear if missing data in high-risk populations occur at random or if there are specific characteristics leading to s*ystematic* missing responses such as missing prompts in particular affective states, social situations etc. (32). Indeed, if one assumes that increasing dysregulation in affect/physiology or psychosocial problems (e.g. interpersonal conflicts) are simultaneously associated with a higher risk of STBs (16) *and* a higher likelihood of missing responses to upcoming prompts, this is highly problematic for monitoring and predicting STBs. Participants with particularly elevated STB risk might have a higher likelihood to miss prompts or drop-out from a study and show, in sum, lower compliance rates than participants that are less burdened. Two studies give some support to the assumption that missing responses do not occur at random. The study of Wang et al. (33) illustrated that missing responses in EMA are relevant predictors of suicide attempts. In the study of Bentley et al. (10) a sensitivity analysis (excluding participants with low compliance) also changed the pattern of results.

Although the risk of reactivity effects due to the repeated assessment of STBs has been discussed repeatedly (specifically regarding iatrogenic effects such as the increase or worsening of STBs) (34), studies systematically addressing measurement reactivity or participant experiences in depth are rare in real-time suicide research (25). So far, there has been no conclusive evidence regarding iatrogenic effects (such as worsening of suicidal thoughts) of repeated assessments (2, 35, 36). Yet, previous studies highlighted that *some* participants report reactivity effects (e.g. 22% worsening of mood and 18% intensifying suicide ideation (25)).

Although several studies have included short self-report questions regarding feasibility/reactivity effects or gave the participants an opportunity to share their views on the study in their debriefings (e.g (26, 37)), qualitative insights illuminating the experiences of participants on reactivity in depth are missing to our knowledge, even though combining quantitative and qualitative methods has been recommended for the analyses of measurement reactivity (38). In suicide research, it might be specifically relevant to incorporate the views of participants that prematurely dropped out from an EMA study or experienced a suicidal crisis/a suicide attempt during the study to understand the potential impact of EMA on these events.

If measurement reactivity is evident, it could seriously limit the ecological validity of the data (39) and generally prevent researchers from implementing intensive EMA study protocols in such a vulnerable population. Consequently, studies examining the effects of measurement reactivity are highly relevant and should in particular address studies employing long observation intervals, with participants with an increased likelihood for unwanted iatrogenic effects and should use qualitative methods to capture the complexity of the experiences.

The present study aims to shed light on measurement reactivity in a long-term EMA study assessing STBs in persons discharged after inpatient psychiatric treatment due to a suicide attempt or suicidal crisis. To fill the described gaps in the literature, we conducted qualitative interviews on experiences of measurement reactivity related to the study with n = 16 participants. The participants differed in terms of study participation characteristics, i.e., high vs. low compliance (with some prematurely dropping out from the study), experiencing no STBs, suicide ideation only or a suicide attempt during the six-month study period, and in terms of gender and age. In order to get novel insights on reactivity effects and feasibility of long-term EMA in a population at high risk of STBs/in persons with lived experiences and to derive recommendations for future studies in the field regarding designing proper study protocols, the following research questions were examined:

− What aspects of measurement reactivity (i.e., changes in experiences and behavior) are described by the participants *because* of the EMAs related to STBs?
− Do the participants suggest any improvements to the study protocol?
− How do the participants evaluate the feasibility of EMA with regard to the long-term monitoring of STBs in general and in times of an acute suicidal crisis in particular?

## 2 Materials and methods

### 2.1 EMA study design

All participants took part in a study examining short-term predictors of STBs following discharge from inpatient psychiatric care due to a suicide attempt/suicidal crisis. The study was preregistered (40, 41) and reporting of the EMA procedures was according recommendations by Trull and Ebner-Priemer (56) Participants were recruited during their inpatient stay in a psychiatric clinic in two study sites. Inclusion criteria were being aged 18–75 years, fluent in German and able to give informed consent. Research staff regularly attended routine meetings in seven clinics in Leipzig and the Ruhr Area around Essen to identify inpatients fulfilling these criteria. Eligible inpatients were approached and informed about the study. After giving written informed consent, a baseline assessment was conducted during which the app “Catalyst” by Metricwire Inc. was installed on their private smartphones. The participants notified the study team when the discharge was scheduled via the app and the EMA surveys started 1 to 3 days prior to discharge and were sent for three weeks after discharge (EMA 1 lasting 21 to 24 days). During EMA 1, the participants received four semi-random prompts between 8:00am and 10:00pm (minimum interval of 120 minutes between prompts). Following initial notification, each survey was accessible for 20 minutes and timed out after that. If ten minutes passed without accessing the survey, a push-notification was sent as a reminder. Each survey consisted of 29 to 31 items on current affect, cognitions and SI rated on a five-point Likert scale from 0 to 4 and two multiple choice items asking about context (see the preregistration for a full list of items (41)). Items were presented in randomized order. Suicide ideation was assessed with three items (*At the moment … I wish I was dead, … I think about killing myself, … I have the intention to kill myself*) in fixed order. If the first SI item was responded to > 0, two branched items were presented asking about suicide-related rumination. Following EMA 1, a second assessment phase started (EMA 2) lasting for 26 weeks. On two random, consecutive days per week the participants received the same EMA protocol and item set. In total, the participants received between 84 and 96 prompts in EMA 1 and 208 prompts in EMA 2. After EMA 1 and EMA 2, STB occurrence was assessed with the Self-Injurious Thoughts and Behaviors Interview via telephone (SITBI-G (42).

### 2.2 Safety protocol and ethical aspects

The app featured information regarding national and regional emergency numbers that were permanently accessible. In EMA 1 the participants received a daily reminder of the emergency numbers, in EMA 2 the reminder was sent once a week. All participants were informed that the study team will not monitor responses and is not providing other than technical support during the study. A crisis card containing emergency numbers was handed out to all participants upon inclusion. During briefing, the participants were informed that repeated EMAs might be experienced as burdensome. They were made aware that they could drop out from the study anytime in case they feel too stressed or have the impression that the EMAs lead to a worsening of symptoms.

All participants gave written informed consent prior participation. All study procedures were approved by the local ethical committees (University of Leipzig/Medical Faculty no. 382/17-ek; University of Duisburg-Essen, no. EA-PSY19/23/03102023) and in accordance with the Declaration of Helsinki.

### 2.3 Compensation and compliance monitoring

In total, the participants could receive up to 170 EUR for their participation: 20 EUR for participating at baseline, plus 20 EUR for participating in EMA 1 and 20 EUR on top for compliance rates > 80%, plus 25 EUR for participating in EMA 2 and 20 EUR on top for compliance > 80%, plus 20 EUR for participating in the follow-up interviews after EMA. Additional 25 EUR were paid for wearing the Polar Unite fitness watch (results not reported here). If they participated in the qualitative interview on reactivity effects, they received additional 20 EUR. Feedback on compliance was continuously available in the app. Reminders to check current compliance levels were sent out every two weeks using standard texts.

### 2.4 Interview participants

It was specified a-priori that participants with different characteristics related to compliance (high vs. low), study completion (completion vs. premature drop-out), STB occurrence during EMA (none, suicide ideation, suicide attempt), gender (male, female) and age should be approached. Between May 2024 and February 2025, study participation ended for n = 76 participants. Of those, 35.5% (n = 27) could be reached and agreed to take part in the final follow-up interview. During the follow-up, research staff check eligibility according the predefined selection criteria. If eligible, they were informed about the interview study and additional informed consent was obtained if they were willing to participate. Sixteen participants were interviewed between May 2024 and February 2025. At both project sites, eight interviews were conducted each (either face-to-face or via an online video call). The resulting sample had a mean age of 39.9 years (SD = 10.1, min = 21, max = 64) and nine (56.3%) identified themselves as male. In EMA 1, ten participants experienced suicide ideation (62.5%) and two (12.5%) attempted suicide. In EMA 2, suicide ideation was reported by eleven participants (68.8%) and three (18.7%) reported to have had a suicide attempt during this time. Please see **Table 1** for further descriptive statistics of the interview sample.

**Table 1:** Sociodemographic/response characteristics and occurrence of STBs for interview participants.

Interview no.	Gender, age	Compliance EMA 1	Compliance EMA 2	Last response	STBs in EMA 1	STBs in EMA 2
1	F, 35–40 years	0.54	0.15	EMA 2, day 17	NA^1^	SI
2	F, 40–44 years	0.62	0.63	EMA 2, day 52	SI/SA	SI/SA
3	F, 40–44 years	0.31	0.06	EMA 2, day 26	SI	SI
4	M, 45–50 years	0.61	0.62	EMA 2, day 52		
5	M, 20–24 years	0.88	0.72	EMA 2, day 52	SI	SI
6	F, 25–30 years	0.71	0.64	EMA 2, day 52	SI	
7	M, 35–40 years	0.91	0.20	EMA 2, day 29	SI	
8	M, 45–50 years	0.64	0.55	EMA 2, day 52	SI	SI
9	M, 35–40 years	0.54	0.15	EMA 2, day 20	SI, SA	SI, SA
10	F, 35–40 years	0.69	0.27	EMA 2, day 51	SI	SI, SA
11	M, 40–44 years	0.88	0.85	EMA 2, day 52		SI
12	M, 60–64 years	0.77	0.87	EMA 2, day 52	SI	SI
13	M, 30–34 years	0.69	0.62	EMA 2, day 48		
14	F, 40–45 years	0.85	0.32	EMA 2, day 31		SI
15	M, 35–40 years	0.23	0.0	EMA 2, day 1	SI	SI
16	F, 50–54 years	0.25	0.61	EMA 2, day 52		

EMA, Ecological Momentary Assessment; STBs, suicide-related thoughts and behaviors; F, female; M, male; SI, suicide ideation; SA, suicide attempt; Careless responses (< 30s from starting to submitting the survey) were set to missing and not included in the compliance rate; participant 5 responded carelessly in EMA 1 (n = 1) and in EMA 2 (n =1), participant 10 in EMA 2 (n = 22) and participant 16 in EMA 2 (n =1). STB occurrence was recorded by the Self-injurious thoughts and behaviors interview – German [SITBI-G (42),] during follow-up at T1 and T2; no SITBI-G was conducted for participant 1 at T1(NA)

### 2.5 Compliance and retention rates in the interview sample

**Figure 1:**
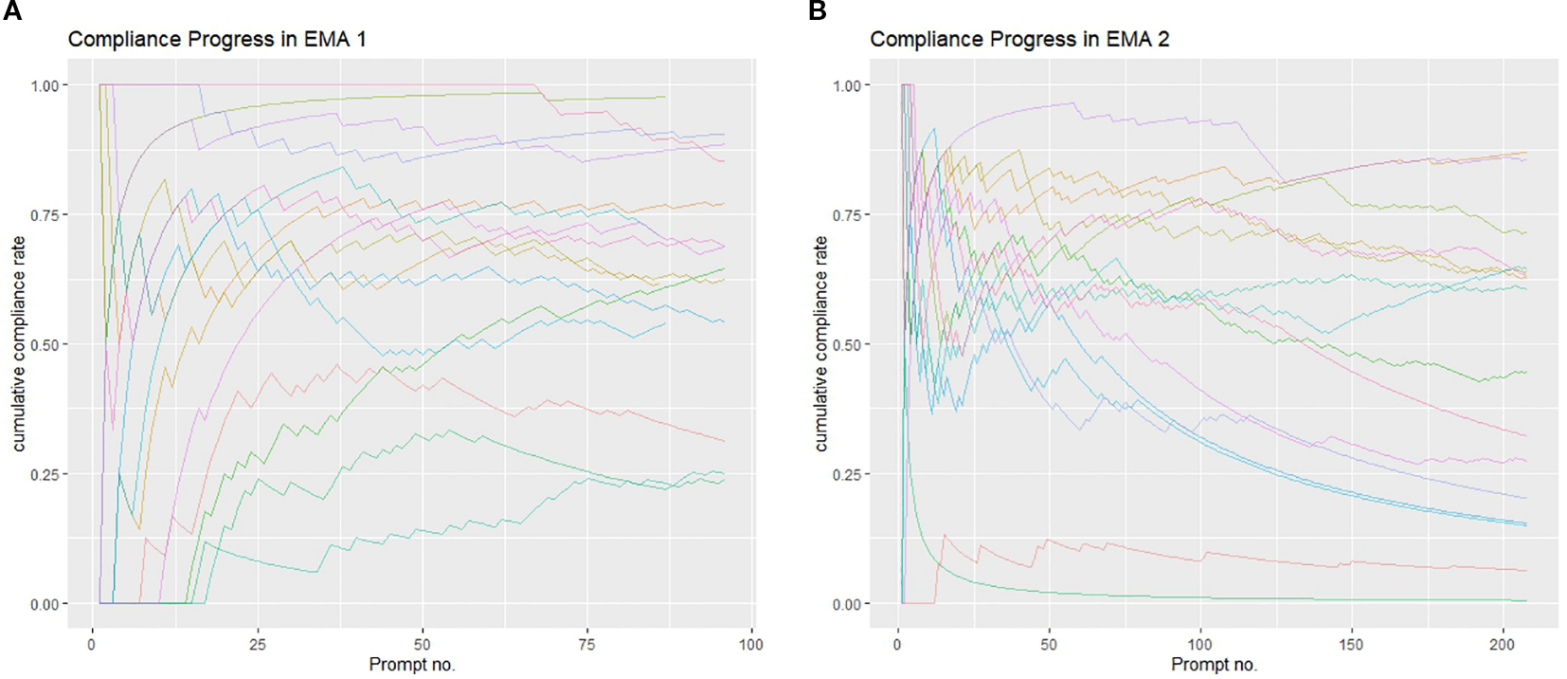
Cumulative compliance. Cumulative compliance over the study phase is plotted on the y-axis (EMA 1 – **(a)**, EMA 2 – **(b)**). For each individual participants one line is plotted (indicated by different colors). On the x-axis, all prompts are displayed ranging from 1 to 96 in EMA 1 **(a)** and 1 to 208 in EMA 2 **(b)**. Steepness of the lines indicate sudden changes in compliances (i.e. several consecutive non responses).

In EMA 1, mean compliance was 63.3% (SD = 21.7, min = 23, max = 91), whereas mean compliance was 45.4% in EMA 2 (SD = 28.5, min = 0, max = 87; see **Table 1**, **Figure 1**). Seven participants stopped participating during EMA 2, i.e., submitted their last response before day 51 or 52). **Supplementary Figures 1**, **2** illustrate the individual response patterns over the course of the study for each participant.

### 2.6 Interviews on reactivity effects and participant experiences

The initial draft of the interview protocol was developed by LS, HG and CS and subsequently discussed by all members of the research team. The interview manual was then presented in a meeting of the qualitative research group of the Department of Medical Psychology and Medical Sociology at the University of Leipzig and several aspects were revised following the recommendations of the group. A pilot interview took place in May 2024 and final adjustments were made.

JS and CS conducted the semi-structured interviews. The interviews were conducted according a field manual (that is provided here (41) in German). In the beginning, participants were reminded about the procedures of the study (i.e. the high-frequency EMA post discharge and the low-frequency EMA later on). All interviewers were instructed to follow a specific communication style (i.e. let participants speak freely, listen actively, use of inviting language and sensory prompts such as “recall a specific moment” to help re-experience situations without suggesting answers). With regard to EMA differentiation,interviewers were asked to distinguish between EMA 1 and EMA 2 (i.e. ask about phases individually and inquire about differences, clarify which phase a statement refers to). The interviews started with an opening question (“Thinking back to that period, what was your personal experience of participating in the smartphone-based surveys?”). If not addressed spontaneously, specific aspects were asked by the interviewers in the following. For example, these referred to general short- and longer-term effects of assessments on mood, feelings, symptoms and thoughts, intensification of suicidal thoughts; feasibility for suicidal crises, integration of assessments in daily routines). All interviews were audio-recorded and transcribed using fx4. The initial transcripts were then manually checked and revised by research assistants.

### 2.7 Qualitative analysis of the interview material

Qualitative Content Analysis (43) was conducted with MAXQDA 24.0 (44) using the following steps: CS, LS and HG developed an initial coding system to answer the preregistered research questions. Two coders (LS, one research assistant) independently coded 20% of the material (i.e., three interviews). During coding, they revised the coding system (i.e., when the initial categories did not capture all the material related to the research questions). LS and the research assistant compared and discussed their coding results and revisions made to the coding system. Collaboratively, they consented a final coding system. Two research assistants then coded all 16 interviews according the final coding system. If further categories came up during the coding process, the coding system was iteratively and collaboratively revised and the interviews were recoded with the consented coding system (see **Table 2**). Results are described according the outlined research questions using 1 to 3 direct quotes per topic/category. Additional quotes are made available in the **Supplementary Material** of the manuscript (**Supplementary Table 1**).

**Table 2:** Final coding system.

List of codes	Frequency of codes
Reactivity: changes in STBs	
(Amplification of suicide plans)	0
Amplification of suicidal thoughts	7
(Amplification of preparatory behavior)	0
Initiation of suicidal thoughts/plans	4
(Initiation of suicidal actions)	0
No changes in STBs	31
Opportunities for improvement	
Study design (items, sampling,…)	46
Study hard- and software (app,…)	12
Study team (contact, support,…)	2
Study information (ICF, …)	5
Feasibility of EMA	
Arguments pro long-term monitoring	27
Arguments contra long-term monitoring	8
Use in suicidal crisis - pro	11
Use in suicidal crisis - contra	13
Time after discharge	11

STBs, suicide-related thoughts and behaviors; ICF, informed consent form; EMA, ecological momentary assessment. Only the codes relevant for the present analysis are included in the table. In the main category “Reactivity: changes in STBs”, three subcategories remained empty (in parentheses).

The qualitative analysis was preregistered before starting the analysis (including the interview protocol in German) and the initial coding systems (40) and reporting followed recommendations for qualitative content analysis (43).

## 3 Results

The final coding system is displayed in **Table 2**. In the following, the content of categories relevant to the research questions is described in more detail. **Supplementary Table 1** contains a selection of further quotes relating to reactivity effects and feasibility of EMA.

### 3.1 Reactivity: changes in suicide-related thoughts and behaviors

With regard to STBs, it was reported that a more differentiated perception of one’s suicidal thoughts increased (e.g., thinking about *or* planning to kill oneself). Additionally, participants reported varying experiences regarding the reactivity of the EMA items on suicidal ideation. Participants reported that the EMA surveys did not affect or interact with their suicidal thoughts and in neither of the interviews it was mentioned that they affected suicidal plans, preparatory behavior or suicidal behavior. On the contrary, the surveys were acting as a reminder for suicidal thoughts and brought them into consciousness instead of intensifying them.

“But otherwise, the questions were more of a reminder of them than they were any kind of amplification.” (int 12).

Indeed, the surveys were sometimes deemed helpful to put the current suicidal thoughts into perspective of a longer time and consequently to decrease them over the study period. However, the opposite effect was reported by others (i.e., that the frequency or the intensity of suicide ideation increased following the EMA surveys). This effect seemed to be more pronounced during periods of intensified suicidal ideation and appeared to vary over time, depending on the individual’s current state or context. The EMA surveys could also initiate suicidal thoughts for some participants, although these effects were reported to be short-lived (i.e., lasting for a few hours).

*“Not often, but it definitely did happen…. That certain thoughts then got introduced. Where I hadn’t been thinking about suicide beforehand, but then despite that started thinking about it after the questions.” (int 7).*

Looking at the frequency of reactivity reports in subgroups of interviews, it appeared that reactivity effects were more often reported in participants with low compliance vs. high compliance.

### 3.2 Ideas to improve study design

The interviews revealed several aspects that could optimize the study. Some were related to study design: To account for familiarity effects, it was recommended to use new items from time to time and to reduce the number of surveys. Since responding to the surveys became more difficult for some participants when getting back in a job, some participants would have appreciated extending the response window or offering the opportunity to postpone a survey. Another aspect that would foster compliance and motivation would have been daily surveys in EMA 2. In addition, it was suggested that the EMA app had more features such as a progress bar and individual feedback on symptom trajectories. This feedback would have been appreciated related to sleep, symptoms and also to the level of suicidal thoughts. The latter was seen as an opportunity to recognize downward spiraling or a decrease in mental health early enough to initiate prevention efforts.

*“Like if I were to imagine, here I am doing it two to three times a week, that I would answer those questions and then had a diagram where I could see, so to* sp*eak, how I had answered, that that would in fact also help me to see: okay, you’re increasingly restless again. You’re more anxious, you’re having more and more suicidal thoughts or something like that – that I could maybe step in for myself more quickly.” (int 14)*.

To enhance safety, it was further suggested to integrate a direct link to a crisis line in the app that is easily accessible.

*“That was a good idea with the emergency numbers, but it was kind of a lot of numbers and maybe just one number like that, yeah, some sort of 24/7 hotline.” (int 11)*.

While the mental state of participants often improved in the months after discharge and symptoms decreased, the item set was not changing and in consequence often not well matching the experiences of participants. Moreover, the assessment of context and activities could have been enriched according to the participants to better reflect the individual situation.

### 3.3 Feasibility: long-term monitoring of STBs

Several aspects were mentioned in favor of long-term monitoring of suicidal thoughts. The EMA surveys generated a feeling of connectedness and support, specifically directly after discharge. The mere existence of the app and the integrated emergency contacts were perceived as helpful and enhanced a feeling of safety. In addition, feelings of loneliness were reduced by the frequent assessments and the app served as support when outpatient care was not accessible right after discharge.

*“And it was in fact kind of stabilizing for me. Like, it really become an everyday companion of sorts. These questions…/So, I’m not currently in outpatient psychological therapy. And it’s difficult for sure to find someone because they’re all totally overrun. So, it actually can be useful and a support even to just have an app that’s feeding you these questions. And you know that somewhere out there are the people who developed it, and that does, yeah that/that helps. It helped me at any rate. So (…) yeah it’s a little support in everyday life, and also having the feeling that you are not as alone.” (int 11)*.

It was also suggested that the regular monitoring increased the sensitivity for downward spiraling and consequently might have increased the likelihood to seek help before a suicidal crisis escalates.

*“That would activate me to do something. Because in that moment, when I see, okay there’s that question about suicidal thoughts, and right now I would click a five. Maybe I should go to my doctor with these strong suicidal thoughts and get some counseling and maybe even take part in another crisis intervention.” (int 6)*.

The EMA surveys even helped to disrupt thinking in circles. However, the monitoring was mainly perceived as useful when suicidal thoughts are generally present and some aspects were speaking against long-term monitoring. In the later phase of the study, the surveys were sometimes perceived as annoying, burdensome, reminders of the past problems and kind of disruptive.

### 3.4 Feasibility: times of high distress and acute suicidal crises

There were mixed opinions if EMAs could be responded to during a suicidal crisis. While it seemed highly likely for some participants to answer regular prompts also in such times, it appeared less likely to self-initiate surveys during a crisis.

“*I believe the approach of having the questions sometimes come regularly and sometimes intermittently is better. (…) in both cases it happened that the suicide attempt was planned over/so, the first time, over multiple days, and the other time over a week and a half or so, and I ended up answering the questions correspondingly, that is, that I am highly suicidal at the moment. But I do believe that to answer then, so proactively then in that phase, that I believe is probably rather unrealistic.” (int 9)*.

The level of self-control/self-regulation seemed to play a role for the likelihood to answer surveys in a crisis, i.e., when alcohol consumption or other factors reducing self-regulatory efforts come into play the likelihood for non-response is higher. On the other hand, it was reported that answering the prompts in times with severe suicidal thoughts created a feeling of pride and helped to regain control over the thoughts. In another interview, the surveys were answered during a crisis but the suicidal thoughts were not altered in either way. Indeed, it was reported that the available emergency contacts encouraged to seek help during a crisis.

Several aspects highlighted a reduced feasibility of EMA during a suicidal crisis. Most importantly, participants considered responding to surveys during a crisis unlikely or have even stopped to do so in a crisis. Reasons for non-response included being in a dissociated state, having a narrow focus (before tunnel vision” int 5) and being annoyed or disturbed by the surveys. It was also mentioned that a suicidal crisis might develop too rapidly and there might be a point where suicidal ambivalence ends and the suicidal intent is too strong, leading to non-disclosure and social withdrawal (i.e., non-response in the EMA).

*“Things do escalate pretty quickly, because it goes from/(…)… (incomprehensible) to planning and then executing, and then there’s not much more room for anything else because, yeah, after that, then nothing nothing matters. So yeah, that’s probably also that, that* sp*iral, that in that moment when you’ve reached the decision, everything is meaningless and then all of a sudden there’s a sense of relief … nothing at all matters anymore, whether I answer the questions or not, whether the world improves or not.” (int 10)*.

*“Yeah that’s what’s fatal about it I think, for someone who’s wanting to do that. On the one hand they feel lonely and don’t have anyone they can talk to or any possibility of exchanging with anyone in that moment when they’re having suicidal thoughts. At the same time, I was very aware that if I tried to talk to my husband about it, then yeah, now you can’t go through with it anymore. So that was the paradoxical thing about it. You don’t want to live anymore. You want to be dead, so it’s kind of a wrong move to go to someone and say ‘I want to die now.’ They’re going to do everything they can to stop it from happening. And in that moment that is in direct contradiction with what I want, which is why, whenever it did come up, I always kept it to myself and never involved others.” (int 12)*.

## 4 Discussion

This study explored participants’ experiences with long-term EMA in the critical time after psychiatric discharge. The qualitative approach offered nuanced insights and revealed some key findings: Some participants reported that EMA increased or triggered suicidal thoughts, though effects were short-lived, highly individual, and not linked to harmful behavior. Despite concerns, EMA was generally seen as feasible and helpful after discharge, supporting self-reflection and symptom monitoring. However, motivation often declined over time, emphasizing the need for adaptive, user-friendly protocols. During suicidal crises, EMA appeared less feasible due to reduced ability or willingness to respond, especially in dissociative or impulsive states which advances our knowledge with regard to these specific situations. This limits its value for real-time risk detection or intervention and complicates the interpretation and predictive use of EMA data. Participants emphasized the importance of integrating safety features (e.g., direct access to help). While EMA may be a useful tool to track the trajectory of suicidal thoughts in clinical care or during high-risk transitions, its potential to prevent suicidal behavior remains uncertain and must be further critically evaluated, particularly in high-risk populations. In the following we will discuss the findings in more detail and in the context of previous findings.

### 4.1 Reactivity: changes in STBs

As found in other studies on participants’ experiences, suicidal thoughts could be reactive to EMA (25). Suicidal thoughts could intensify or even be initiated by EMA assessments. While this contrasts with previous studies on reactivity effects in suicide research (36, 45), the nature of our sample and the sampling protocol might offer an explanation for this discrepancy. It has been suggested that individuals with more severe psychopathological symptoms might be more reactive to EMA (29) and we did examine persons with a particular high-risk of STBs in the present study. Yet, the effects appeared to be very individual (i.e. some participants reported such an effect while others did not). It is worth noting, that despite the reported increase in suicidal thoughts, this effect appeared not to be harmful (i.e., leading to harmful behavior). There were no reports that the EMA surveys exerted an impact on planning or suicidal behavior. It remains unclear if the briefing instructions (i.e., stopping EMA in case of being too burdened/experiencing worsening of one’s mental health) might also play a role for this finding or if indeed, the reactivity effects are weak in size. Quantitative approaches have not found evidence of systematic reactivity effects so far (36, 45) which could partly be explained by the characteristics of the reactivity reports in our qualitative data: The effects were short-lived (fading out after few hours) and thus lagged analysis (i.e., prompt to prompt) might not be able to capture them in the case of longer intervals between prompts. Simple linear models looking at a general increase or decrease over time might also miss reactivity effects since the reactivity seemed to depend on the current level of suicidal thoughts (e.g., higher momentary suicidal ideation appeared to be more reactive to surveys).

### 4.2 Improving studies

The interviews revealed several aspects that can help to improve the design of EMA studies and thus foster motivation and compliance during a long-term study. The findings highlight the crucial role of the sampling protocol and the number of items, which has already been advocated for by numerous researchers (46). According to the findings it might be worthwhile balancing the number of positive and negative items and favoring an easy-to-understand sampling protocol (such as a combination of daily diaries and microbursts) over a more complex scheme. In suicide research, the unpredictable occurrence of STBs and their often-sudden increase (or decrease) (15, 16) complicate the development and realization of protocols that capture the phenomenon of interest in the long-term. Participants experience different trajectories related to their symptoms and changing demands related to their personal life calling for more adaptive sampling protocols (e.g (47, 48). This could positively affect compliance and retention but poses challenges regarding the analysis of the data and would require pilot studies how and when adaptive sampling could be realized in that population. The features most suggested by participants are nowadays easily implementable in the newest EMA apps such as provision of a symptom feedback or progress bar (49). Providing feedback might, however, be an intervention on its own right as well (50), which can be useful for clinicians and patients but might interfere with the research questions under study. Based on our findings, the safety protocol of EMA studies on STBs should ensure that the apps feature a low-threshold and easily accessible option to get help (such as directly calling a crisis line). If possible, it might be also a promising approach to additionally enter individualized numbers where participants may seek help in an upcoming crisis (i.e., psychiatric clinic, close friend or family member).

### 4.3 Feasibility of long-term monitoring

The participants mentioned several benefits of the EMA-monitoring that supported its feasibility after discharge. They referred to the EMA surveys as a helpful tool to continuously self-evaluate symptoms and mood and felt supported by the app and the study team behind it. In a way, this appeared to be a substitute of professional care that is not instantly accessible. Since the time following discharge carries a particular risk for STBs (21, 22) and the ease of the transition is facilitated by aspects such as supported autonomy and safety (51), EMA monitoring could be specifically useful during that time and future studies should examine if monitoring has a similar impact on readmission rates as outpatient care has (52). Participants have pointed out that the monitoring could increase the likelihood of early detection, disruption of worsened suicidal thoughts and even help-seeking behavior. Yet, since the benefit of the monitoring fades out for some individuals when their mental health improves and can even lead to the opposite (e.g., burden, annoyance), it remains difficult to establish an adequate length of the monitoring (i.e., how long should long-term be?). In light of the individual trajectories of STBs in the context of factors such as loss of housing or job, partnership problems, relapse related to substance use, somatic illness or others it appears almost impossible to find a one-size-fits-all solution. The potential of EMA is here connected to its ability to adapt to the needs of the individual which has been recently suggested (13). It might be an option to discuss this tailoring openly with the participants when starting the long-term monitoring and to provide different opt-out scenarios for the monitoring for weeks and months.

### 4.4 Feasibility of EMA during a crisis

In the interviews, mixed experiences regarding the feasibility of EMA in times of high distress such as in a suicidal crisis emerged. If at all, regular prompts appeared superior compared to the idea of event-contingent prompts (that is to self-initiate a survey when a crisis develops). One participant who had attempted suicide twice during the study had answered the questions accordingly (i.e., stated that he had a suicide plan prior to the attempts), which is in line with one case study (53) and shows that monitoring responses and reaching out might prevent an attempt.

Yet, the likelihood for non-response during a suicidal crisis appears high. Two aspects contribute to this finding, i.e., a reduced ability and a reduced willingness to respond during a crisis. The reduced ability to answer to prompts was related to decreased levels of self-regulation/self-control or loss of reality such as dissociation; answering prompts contrasts with these dysregulated states. In line with the Dual-System-Model of Suicidality (16), it seems possible that EMA can be feasible for planned suicide attempts but not for suicidal actions driven by the impulsive system. However, if participants know they are monitored and that actions will be taken, planned non-response might also occur for planned suicidal actions. Persons with decreased suicidal ambivalence often choose not to disclose suicidal thought and plans to avoid interference with their actions and non-disclosure of suicidal ideation is higher among those who die by suicide (54). Missingness has indeed been shown to predict suicide attempts in one study (33). Unfortunately, non-response can also occur in the context of other events – such as losing or switching a phone, study fatigue or starting a job where smartphones are not allowed etc. – and is most likely not very specific (i.e., suffers from low predictive accuracy as most of the STB risk-factors do, too) (17). Taken together, non-response can indicate a suicidal crisis but might also reflect other disturbances. It seems advisable to reach out to participants by calling them or send a reminder of emergency numbers as part of the safety protocol if researchers have the resources to do so. The likelihood of non-response in a crisis is seriously undermining the potential of EMA to detect moments and preceding states of increased risk in studies and makes it questionable if EMIs could aid in preventing suicidal actions on a larger scale (13, 14). Even if other markers such as physiological variables or passive sensing data will be identified as short-term risk factors, it is unknown if EMIs reach the target persons or will be used by them.

### 4.5 Strengths and limitations

Major strengths of the study include the heterogeneous nature of the sample and the qualitative approach. While the participants were all at high risk of STBs, they performed differently in the study and varied regarding sociodemographic characteristics as well as the occurrence of STBs during the study period. Including persons with lived experience (i.e., that have actually attempted suicide during an EMA study) creates novel insights on the concerns about iatrogenic risks and was lacking in studies addressing reactivity effects and feasibility of EMA in suicide research (25). The semi-structured interviews revealed more various and multifaceted experiences of participants than debriefings or rating scales (55). Yet, we have not contrasted the subjective reports on reactivity effects with quantitative data which limits the interpretation of the findings. Despite the inclusion of participants who dropped out from the study, our sample might nonetheless suffer from a selection bias limiting also the generalizability of the findings. Participants who agreed to take part in the interview were presumably more functional and less severely burdened than participants who were lost at follow-up.

### 4.6 Conclusion

Our findings provide support for the recommendation that reactivity effects should be examined using mixed-methods approaches (38). Qualitative data in the present study has demonstrated that STBs can be reactive to EMA and future quantitative analyses can be guided by the nature of the reported effects (e.g., being short-lived, situation-dependent). In light of the individual nature of reactivity effects, combining nomothetic and idiographic approaches seems promising. Our work underlines the importance of extensive piloting in clinical populations with higher psychopathological burden and to develop sampling protocols using participative approaches (46) to improve feasibility and thus data quality in this field. Still, coming up with feasible sampling protocols for longer-term assessments remains challenging due to the dynamic and individual trajectories of STBs in the long-term.

Given the critical perspective on the feasibility of long-term monitoring and use during suicidal crises, the potential of EMA/EMI for detecting and preventing suicidal behavior must also be critically seen. In fact, one of the major problems in suicide research re-emerged (i.e., identifying the moment of sudden risk increase in a clinically relevant time frame of hours and days with high predictive accuracy (17)). Missingness in the data is a particularly problematic issue here, since it appears to occur not at random, but often relates to a reduced ability or willingness to respond to EMA surveys. The usefulness of EMA might lie specifically in the lower end of the suicidal spectrum, that is to aid in understanding the occurrence and maintaining factors of (individual) suicidal thoughts and might be a particularly useful tool to monitor their trajectories and related states and symptoms. In particular, this might be of use in the transition between inpatient and outpatient care or in clinical treatment of suicidal thoughts.

## Data Availability

The raw data supporting the conclusions of this article will be made available by the authors, without undue reservation.
