# Supplementary material for: Qualitative content analysis of reactivity effects and feasibility of ecological momentary assessments of suicide-related thoughts and behaviors in the long-term and in suicidal crises
Source: Front Psychiatry. 2026 Mar 6;17:1744947. doi: 10.3389/fpsyt.2026.1744947 (PMC13003599; doi:10.3389/fpsyt.2026.1744947)
Supplement: Supplementary file 1 [file DataSheet1.pdf]

Supplementary material

Compensation scheme:

All participants could receive the following incentives:

- 20 EUR for participating at baseline,
- 20 EUR for participating in EMA 1 and 20 EUR on top for compliance rates > 80%,
- 25 EUR for participating in EMA 2 and 20 EUR on top for compliance > 80%
- plus 20 EUR for participating in the follow-up interviews after EMA.
- Additional 25 EUR were paid for wearing the Polar Unite fitness watch (results not reported here).

If they participated in the qualitative interview on reactivity, they received additional 20 EUR.

Supplemental Table S1: Selection of quotes relating to reactivity and feasibility of EMA

**Reactivity: Changes in STBs**	
Changes in suicidal thoughts	*Amplification or intensification*<br>"It didn't lead to me becoming active in any way to try to attempt anything. But it did serve as a kind of confirmation of sorts for me. Like, yes, that would be the way to do it." int 5<br>*Short-lived nature*<br>"Mostly, I thought about it for one, two, three hours afterward. Not whether I wanted to do it or how I want to do it, but just thinking about it in general and then it eventually evaporated at some point." int 7<br>*Interaction with current state or context*<br>"Yes [suicide ideation intensified], that is, at least in the really bad phases." int 14<br>"In my particular case it was true that I then simply, as I said, after the suicide attempt, I noticed I can't actively deal with it right now. That's the very thing I have to get some distance from, from those thoughts. And if I were to answer those particular questions in that very moment, then it would really not be helpful for my recovery process at that time. In the period before that though, it wouldn't have been upsetting at all."int 9<br>*Decrease*

	“I would say that they definitely decreased because even in moments when I was worse off I would think to myself/or I also knew that some days are better than others. And that it’s possible, even for me, to feel much better. And that just because I feel terrible right now, it doesn’t mean that I always feel terrible. And that definitely helped with reigning things in a bit.” int 5
Initiation of suicidal thoughts/ plans	“Yes, so that’s what I mean by triggering. So I definitely did have moments where I was really really really fed up, where one in fact does end up following certain thoughts down that path. Yeah, that has happened to me, it’s happend to me before. I mean, not such that it actually led to a long active thought process, but yes, things definitely got triggered in that direction.” int 14
	No effect (when asked if EMA surveys have had an impact on STBs)
	“I mean, I really do have the feeling that there was no interaction between the effect of the survey and how I felt.” int 8
	"In general, I was very open about the topic [SI] with my friends and the people around me. I talked about it openly, so for me, answering the questions didn’t have any effect—it wasn’t a big deal." int 9
No changes in STBs	“Looking back, it actually turned out to be a good thing that I dealt with the symptoms more closely. But they didn’t change right away because of that.” int 2
	Reminder of suicidal thoughts
	“The suicidal thoughts were usually already there. So, no they weren’t prompted by the questionnaire, rather, when I did click ‘yes, suicidal thoughts are present,’ then it was because they had already been there before the question came up.” int 6
**Feasibility – long-term monitoring**	
	I mean, for one thing, simply the reminders with the emergency numbers and just having the app at all gives one a certain sense of security.” int 4
	“Personally, I think I could recognize more quickly if things are somehow taking a turn for the worse because that is in fact kind of a problem, somehow a problem that, in my experience, all depressed people have in common, that we think for too long: Hey, We’ve got this. It’s not that bad. And then wait too long to get help. Either because we don’t want to recognize it, or in fact really just aren’t able to recognize it because we don’t take the time to listen to ourselves and consider: What is really going on right now?” int 14
Pro	“But in both phases it had the same effect then, that it kind of pulled me out of the cycle [of thinking about suicide] a little.” int 14
	“that, for example, if someone says, I wish were dead right now, that in that situation, really, regardless of whether someone is constantly thinking about it or not, that at that time there was already a certain/ It would be good, if there was any kind of tracking there at all.” int 12
Contra	“At the end of the survey it did start to be a bit (...) of a nuisance, I’d like to say. Simply because it just so happened to be Easter at the time, and I in fact really just didn’t like remembering in any way what a bad phase I had been through, and I was doing better at the time and therefore experienced it as being a bit oppressive. But like I said, that wasn’t until the very end of the survey period.” int 2

	"... At first it was very positive and it also helped me to just generally reflect again and be more conscious about my whole situation. And then once I had gotten over the worst of it, so to speak, it of course did serve as something of a negative reminder of how bad of a phase I had had." int 2<br>That at some point it simply was too much for me. Like, like, this whole thing of thinking about "How are you actually doing?" and especially in relation to suicidal thoughts, once that sort of recedes into the background, that was more/ well that I did actually experience as being kind of a burden. int 1
**Feasibility – suicidal crisis**	
Pro	"Well, for me it would be possible" int 4<br>"The chances are fifty/fifty I would say. It's dependent on all of the circumstances and everything, but (...) yes, as long as I'm not currently under the influence of alcohol or anything then it's easy to remember. As soon as various other influences come into play, it starts to get more and more difficult. Then all of a sudden it's just here and there." int 7<br>I was proud of myself that I was able to do it. And simultaneously distracted from the thoughts. In that moment then they were supressed." int 16<br>"I continued responding at that point too,... That one then starts to ask themselves, where/ '(is it getting?) worse, should something change?' And if suicide might be a way of figuring that out, whether a suitable one or not. One does think about it, but it's not the questions that are the trigger. At least not for me." int 3<br>"For me it was good to receive the notifications with the hotline numbers. And, um. (...) over the course of the whole thing I did end up using one once, so based on that, it really does make sense." int 2
Contra	"I did end up having another crisis in the meantime. And in that case, true, I forgot about that, that I told your colleague about it too. At that point, I was in such bad shape then that I just quit responding. ... because I just didn't care anymore, because things were strange, and I wasn't myself anymore. Yeah, I just/ Well, everything that happened then wasn't real in the moment and the other way around, because I simply wasn't aware of it anymore. Like, because I just wasn't doing it anymore..." int 8<br>"I think in a situation like that that it would probably annoy me or/ but because one kind of tends to have a bit of tunnel vision focused on a single thing and then you get this notification 'the questions are ready.' and that can actually be kind of a nuisance. One feels, I don't know, you kind of feel like just chucking the phone and clock and keeping completely to yourself." int 5

Supplemental Figure 1: Response patterns in EMA 1

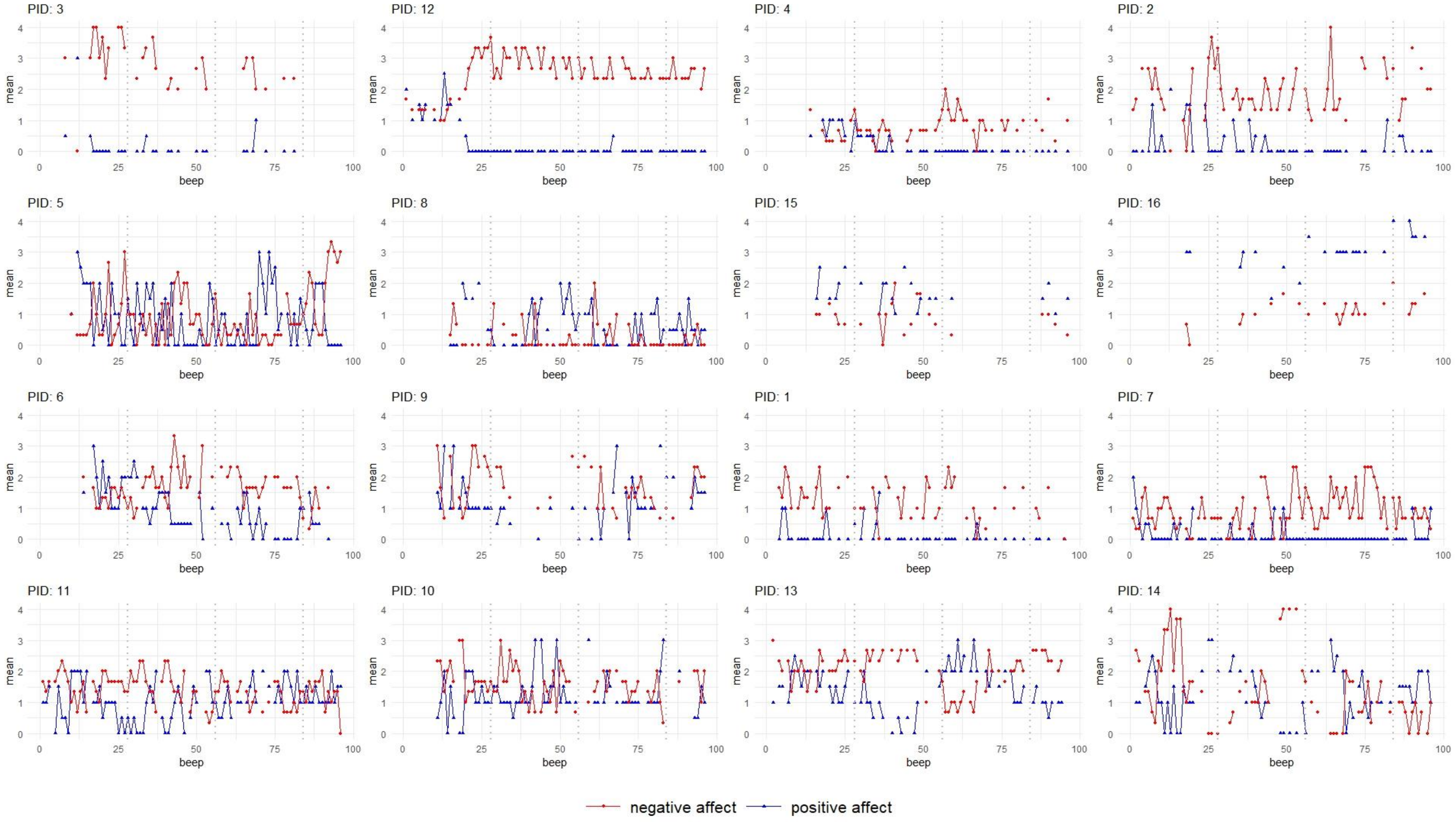

Notes: Individual trajectories for all 16 participants are plotted (using R packages "ggplot2" (Wickham 2016) and "patchwork" (Pedersen 2024). Observations are displayed on the x-axis (ranging from 0 to 96, i.e. four prompts on 21 to 24 days. The mean of three negative affect items ("downhearted", "sad", "anxious") and two positive affect items "happy", "cheerful") are displayed on the y-axis (all items were rated on a four point Likert scale with means ranging from 0 to 4). Missing values are not displayed.

Supplemental Figure 2: Response patterns in EMA 2

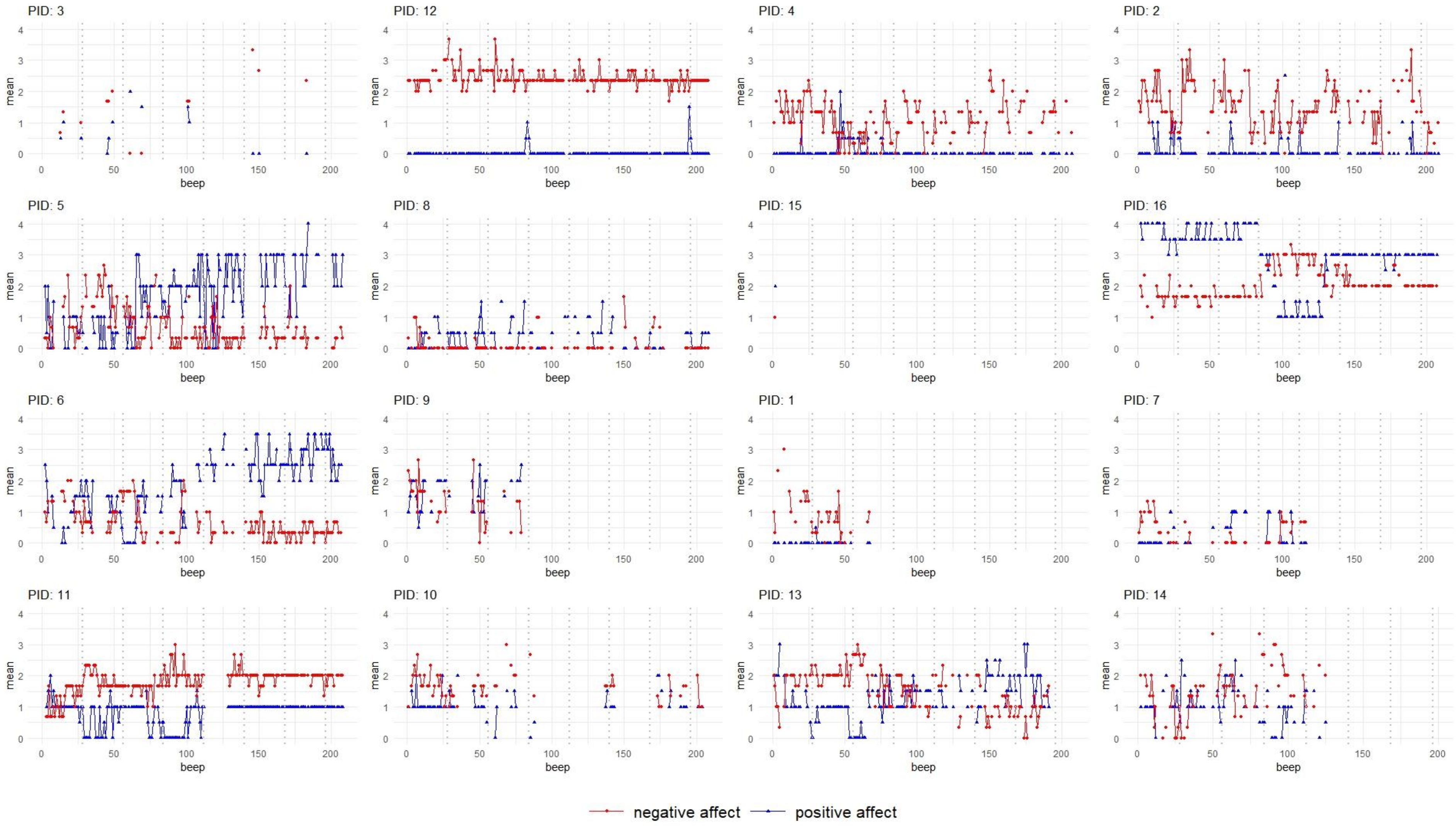

Notes: Individual trajectories for all 16 participants are plotted (using R packages "ggplot2" (Wickham 2016) and "patchwork" (Pedersen 2024). Observations are displayed on the x-axis (ranging from 0 to 208, i.e. four prompts on 52 days sent over 26 weeks. During each week, the participants received prompts on two randomly chosen consecutive days. The mean of three negative affect items ("downhearted", "sad", "anxious") and two positive affect items "happy", "cheerful") are displayed on the y-axis (all items were rated on a four point likert scale with means ranging from 0 to 4). Missing values are not displayed
